# Supplementary material for: Wound Healing, Antioxidant, and Antiviral Properties of Bioactive Polysaccharides of Microalgae Strains Isolated from Greek Coastal Lagoons
Source: Mar Drugs. 2025 Feb 10;23(2):77. doi: 10.3390/md23020077 (PMC11857774; doi:10.3390/md23020077)
Supplement: Supplementary file 1 [file marinedrugs-23-00077-s001.zip › marinedrugs-3425452-supplementary.pdf]

Supplementary Materials

Wound Healing, Antioxidant, and Antiviral Properties of Bioactive Polysaccharides of Microalgae Strains Isolated from Greek Coastal Lagoons

Gabriel Vasilakis [1,†], Sofia Marka [2,3,†], Alexandros Ntzouvaras [2,4], Maria-Eleftheria Zografaki [2], Eirini Kyriakopoulou [5], Katerina I. Kalliampakou [2,5], Georgios Bekiaris [1], Evangelos Korakidis [5], Niki Papageorgiou [2], Stefania Christofi [1], Niki Vassilaki [5], Georgia Moschopoulou [3], Ioannis Tzovenis [4], Athena Economou-Amilli [4], Seraphim Papanikolaou [1] and Emmanouil Flemetakis [2,*]

[1] Laboratory of Food Microbiology and Biotechnology, Department of Food Science and Human Nutrition, Agricultural University of Athens, 11855 Athens, Greece; vasilakis.gavriil@gmail.com (G.V.); giorgosbekiaris@yahoo.gr (G.B.); christofistefania@gmail.com (S.C.); spapanik@aua.gr (S.P.)
[2] Laboratory of Molecular Biology, Department of Biotechnology, Agricultural University of Athens, 11855 Athens, Greece; smarka@aua.gr (S.M.); alexntzouv@gmail.com (A.N.); mzografaki@aua.gr (M.-E.Z.); kalliamp@yahoo.gr (K.I.K.); nikipapageorgiou99@gmail.com (N.P.)
[3] Laboratory of Cell Technology, Department of Biotechnology, Agricultural University of Athens, 11855 Athens, Greece; geo_mos@aua.gr
[4] Sector of Ecology & Systematics, Department of Biology, National and Kapodistrian University of Athens, 15784 Athens, Greece; itzoveni@biol.uoa.gr (I.T.); aamilli@biol.uoa.gr (A.E.-A.)
[5] Laboratory of Molecular Virology, Hellenic Pasteur Institute, 11521 Athens, Greece; eirhnh63@gmail.com (E.K.); korakidis13@gmail.com (E.K.); nikiv@pasteur.gr (N.V.)
* Correspondence: mflem@aua.gr; Tel.: +30-21-0529-4343
† These authors contributed equally to this work.

Table S1. Growth parameters of the various microalgae strains used in the present study. Starting cell density (CD_s), final cell density (CD_f), growth rate during exponential phase (μ_{max}) and final dry biomass (X) concentration after 10 days of cultivation of microalgae on using Walne's culture medium under specific conditions.

Strain	CD_s (cells/mL x 10^5)	CD_f (cells/mL x 10^5)	μ_{max} (d^{-1})	X (g/L)
Tetraselmis verrucosa f. *rubens* PLA1-2	0.90 ± 0.01	17.70 ± 0.51	0.675 ± 0.010	0.564 ± 0.162
T. suecica T3-1	0.88 ± 0.02	13.90 ± 0.14	0.698 ± 0.054	0.394 ± 0.119
T. verrucosa f. *rubens* KSI1-3	1.10 ± 0.04	13.70 ± 0.14	0.502 ± 0.027	0.882 ± 0.044
T. suecica ELO1-1	0.95 ± 0.02	24.70 ± 0.76	0.658 ± 0.033	1.060 ± 0.113
Tetraselmis spp. Mes5	0.81 ± 0.03	5.24 ± 0.28	0.520 ± 0.014	0.497 ± 0.083
Dunaliela salina 32	0.84 ± 0.02	4.89 ± 0.21	0.355 ± 0.020	0.240 ± 0.049
Tetraselmis spp. Mes17	0.82 ± 0.02	4.85 ± 0.21	0.436 ± 0.021	0.461 ± 0.041
T. suecica ELO1-2	0.93 ± 0.04	11.56 ± 0.43	0.570 ± 0.037	0.894 ± 0.034
T. verrucosa f. *rubens* KLE1-3	1.17 ± 0.04	14.56 ± 0.43	0.532 ± 0.029	0.805 ± 0.007
Nannochloropsis gaditana PLA1-1	6.51 ± 0.12	141.32 ± 8.65	0.715 ± 0.041	0.893 ± 0.182
D. salina 30	0.81 ± 0.02	5.26 ± 0.17	0.422 ± 0.023	0.208 ± 0.022
T. verrucosa f. *rubens* KLE1-4	0.94 ± 0.06	6.97 ± 0.31	0.461 ± 0.029	0.414 ± 0.002
D. salina 31	0.92 ± 0.03	5.63 ± 0.19	0.385 ± 0.034	0.240 ± 0.019
Tetraselmis spp. EST1-2	0.95 ± 0.02	8.60 ± 0.48	0.552 ± 0.037	0.538 ± 0.028

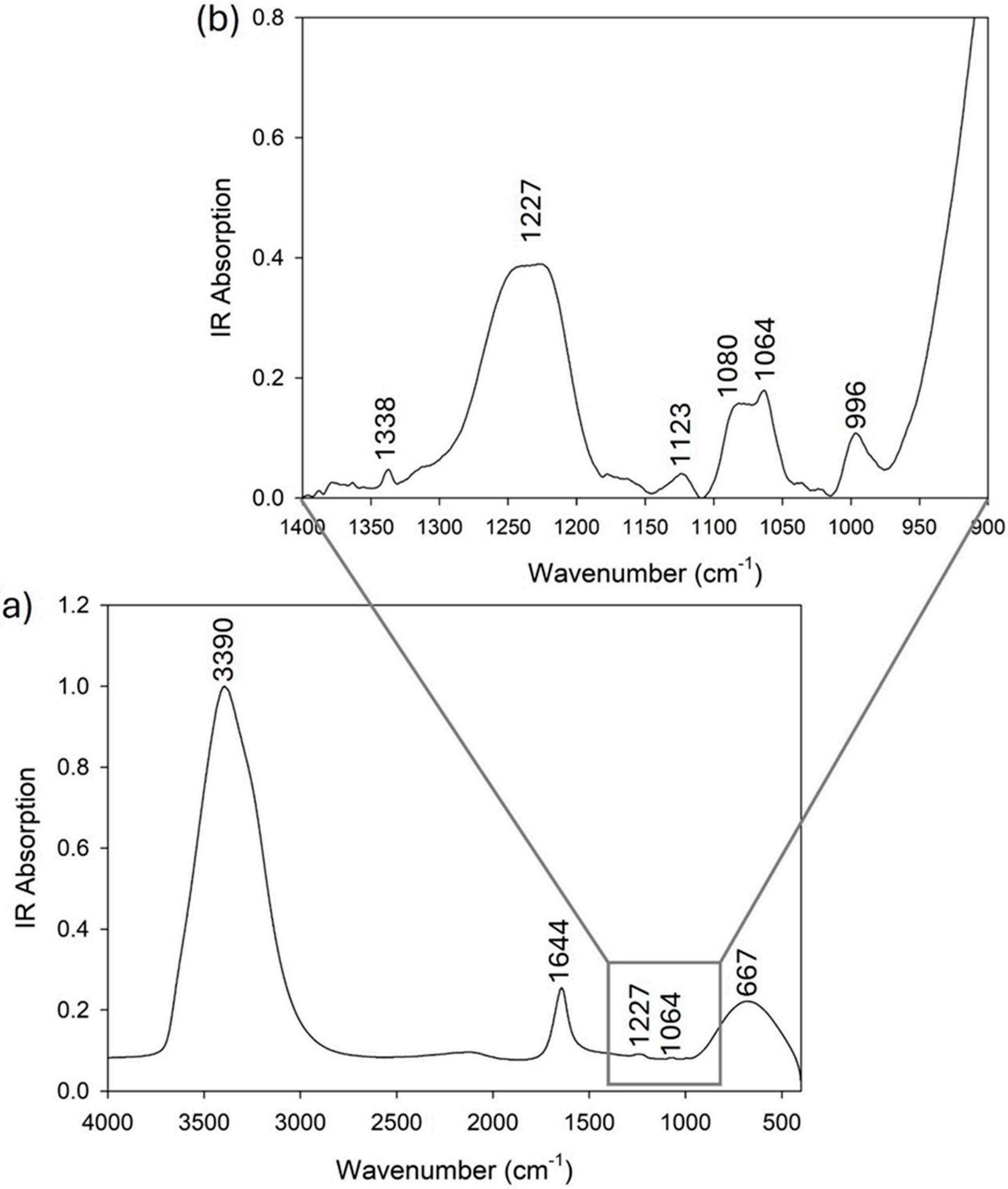

Figure S1. ATR-FTIR spectrum of the *Tetraselmis verrucosa* f. *rubens* PLA1-2 extracts at (**a**) 4000-400 cm^{-1} and (**b**) 1400-900 cm^{-1}. The peaks of interest are marked.
